# Comparison between cephalometric measurements using digital manual and web-based artificial intelligence cephalometric tracing software

**DOI:** 10.1590/2177-6709.27.4.e222112.oar

**Published:** 2022-08-15

**Authors:** Gökhan ÇOBAN, Taner ÖZTÜRK, Nizami HASHIMLI, Ahmet YAĞCI

**Affiliations:** 1 Erciyes University, Faculty of Dentistry, Department of Orthodontics (Talas/Kayseri, Turkey).

**Keywords:** Artificial intelligence, Automatic cephalometric analysis, Digital cephalometric analysis, Skeletal malocclusion

## Abstract

**Objective:**

The aim of this study was to compare the measurements performed with digital manual (DM) cephalometric analysis and automatic cephalometric analysis obtained from an online artificial intelligence (AI) platform, according to different sagittal skeletal malocclusions.

**Methods:**

Cephalometric radiographs of 105 randomly selected individuals (mean age: 17.25 ± 1.87 years) were included in this study. Dolphin Imaging software was used for DM cephalometric analysis, and the WebCeph platform was used for AI-based cephalometric analysis. In total, 10 linear and 12 angular measurements were evaluated. The paired *t*-test, one-way ANOVA test, and intraclass correlation coefficient tests were used to evaluate the differences between the two methods. The level of statistical significance was set at *p*< 0.05.

**Results:**

Except for SNB, NPog, U1.SN, U1.NA, L1-APog, I/I, and LLE parameters, all other parameters presented significant differences between the two methods (*p*< 0.05). While there was no difference (*p*> 0.05) in both SNA and SNB measurements between the two methods in the Class I malocclusion group, there was a difference between both methods in the Class II malocclusion group. Meanwhile, only the SNA in the Class III malocclusion group was different (*p*< 0.05). The ANB angle differed significantly in all three malocclusion groups. For both methods, all parameters except CoA and CoGn were found to have good correlation.

**Conclusion:**

Although significant differences were detected in some measurements between the two cephalometric analysis methods, not all differences were clinically significant. The AI-based cephalometric analysis method needs to be developed for more specific malocclusions.

## RESUMO

**Objetivo:**

Comparar as medidas realizadas usando análise cefalométrica digital-manual (DM) e análise cefalométrica automatizada por meio de uma plataforma online de inteligência artificial (IA), segundo as diferentes más oclusões esqueléticas sagitais.

**Métodos:**

Foram incluídas radiografias cefalométricas de 105 indivíduos selecionados aleatoriamente (idade média: 17,25 ± 1,87 anos). O *software* Dolphin Imaging foi utilizado para análise cefalométrica DM, e a plataforma WebCeph foi utilizada para análise cefalométrica baseada em IA. No total, foram avaliadas 10 medidas lineares e 12 angulares. O teste*t* pareado, a ANOVA de uma via e o coeficiente de correlação intraclasse foram utilizados para avaliar as diferenças entre os dois métodos. O nível de significância foi estabelecido em *p*<* *0,05.

**Resultados:**

Com exceção dos parâmetros SNB, NPog, U1.SN, U1.NA, L1-APog, I/I e LIE, todos os outros parâmetros apresentaram valores significativamente diferentes entre os dois métodos (*p*<* *0,05). Enquanto não foi encontrada diferença (*p*>* *0,05) nas medidas SNA e SNB entre os dois métodos no grupo má oclusão de Classe I, foi encontrada diferença entre os métodos no grupo má oclusão de Classe II. Entretanto, no grupo má oclusão de Classe III somente o SNA foi diferente (*p*<* *0,05). O ângulo ANB diferiu significativamente em todos os três grupos de más oclusões. Foi encontrada boa correlação entre os dois métodos para todos os parâmetros, exceto para CoA e CoGn.

**Conclusão:**

Embora para algumas medidas tenham sido detectadas diferenças significativas entre os dois métodos de análise cefalométrica, nem todas as diferenças foram clinicamente significativas. O método de análise cefalométrica baseado em IA precisa ser aperfeiçoado, com maior especificidade para cada má oclusão.

## INTRODUCTION

Cephalometric analysis performed using certain anatomical points on lateral cephalometric radiographs is an important tool that orthodontists use to plan their treatment and to monitor the development of growing individuals.^1^ ^-^ ^3^ In the past, drawings used for lateral cephalometric analysis were performed manually on transparent tracing paper by orthodontists.^4^ With the development of technology, these processes began to be carried out digitally, and significant improvements were achieved in terms of speed, quality, and reliability.^5^ The digitization of cephalometric images facilitates the treatment planning phase, by eliminating the human error inherent in the stage of traditional X-ray radiographs preparation in dark rooms, and introducing the possibility of digitally storing and sharing images in a practical way.^1^ ^,^ ^5^ ^,^ ^6^ Converting from a manual cephalometric analysis technique to a digital cephalometric analysis technique provides many advantages, but still results in time wasted in front of a computer screen and requires professional supervision.^5^ ^,^ ^7^ ^,^ ^8^ Today, diagnostic procedures based on computer-aided artificial intelligence (AI) are increasing, especially in dentistry applications that require radiographic evaluation.^7^ ^,^ ^9^ ^,^ ^10^ In several studies, it has been stated that AI-based applications are useful in determining the points used in cephalometric analysis, and can be used for measurements based on cephalometric analysis.^3^ ^,^ ^7^ ^,^ ^11^ For these reasons, studies on the reliability and usability of AI still provide insufficient evidence, and it is believed that research into more specific areas should be increased.

The clinical use of information technology in Orthodontics has increased significantly in recent years. Thus, the aim of this study is to compare AI-based cephalometric analysis and digital-manual-based cephalometric analysis (DM) in Orthodontics, and to evaluate the reliability of AI in different sagittal skeletal malocclusion classes.

## MATERIAL AND METHODS

This study, conducted according to the principles of the Helsinki Declaration, was approved by the Erciyes University Clinical Research Ethics Committee (approval no: 2020/498). Prior to the study, informed consent was obtained from all participants and parents/guardians included in the study. In order to determine the results that could produce a significant difference in the study, according to the power analysis using G*Power (v. 3.0.10, Franz Faul, Universität Kiel, Germany) software, it was determined that a 0.05 significance level, 0.85 effect size, and sample size of 35 individuals for each group in 95% power would be sufficient.^12^ Inclusion criteria of the study comprised the following: (1) individuals with dental and skeletal Class I, II, or III malocclusion, (2) patients with ideal pretreatment diagnosis and records, (3) patients without any congenital anomaly or dentofacial syndrome, and (4) cephalometric measurements taken before treatment. It was planned to select individuals with radiographs of adequate clarity and quality. The exclusion criteria were determined as: (1) insufficient diagnosis or inadequate individual records, (2) presence of any congenital anomaly or dentofacial syndrome, and (3) cephalometric radiographs taken before treatment whose inadequate clarity and quality made them unusable. Dolphin Imaging cephalometric analysis software (v. 11.5, California, USA) was used for DM analysis. A web-based, free online cephalometric analysis service called WebCeph (WEBCEPH™, Artificial Intelligence Orthodontic & Orthognathic Cloud Platform, South Korea, 2020) was used for AI-based automatic cephalometric analysis.

**Figure 1:**
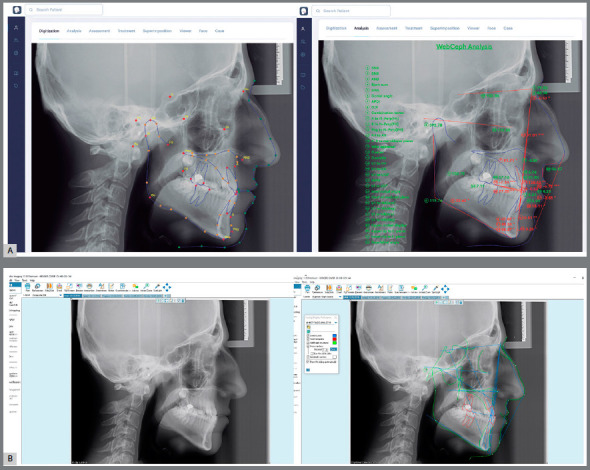
A) Dolphin digital cephalometric analysis program, traditionally used by orthodontists. B) Artificial intelligence-based online WebCeph cephalometric analysis software.

**Figure 2:**
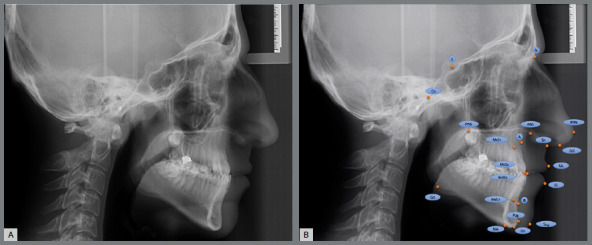
A) Lateral cephalometric radiograph. B) Cephalometric landmarks. S= Sella, N= Nasion, Co= Condylion, ANS= Anterior Nasal Spine, PNS= Posterior Nasal Spine, PRN= Pronasale, A= A point, B= B point, Sn= Subnasale, Col= Columella, Mx1r= Maxillary first incisor root, Mx1c= Maxillary first incisor crown, UL= Upper Lip, LL= Lower Lip, Md1c= Mandibular first incisor crown, Md1r= Mandibular first incisor root, Go= Gonion, Pog= Pogonion, Me= Menton, Gn= Gnathion, SPog= Soft tissue pogonion.

**Figure 3:**
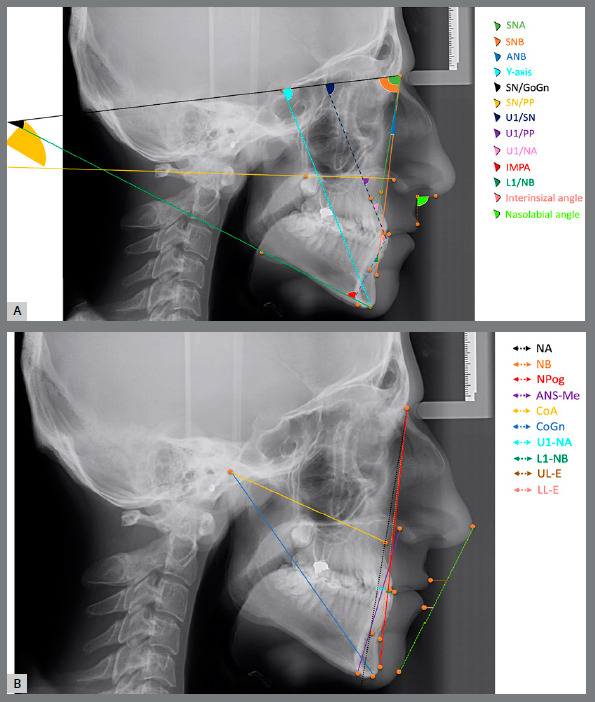
A) Linear measurements; NPog= Nasion?Pogonion, NA= Nasion?A point, ANS-Me= Anterior Nasal Spine-Menton, CoA= Condylion-A point, CoGn= Condylion-Gnathion, U1-NA= Long axis of upper incisor?Nasion-A point, L1-APog= Long axis of lower incisor?A point-Pogonion, L1-NB= Long axis of lower incisor?Nasion-B point, ULE= Distance of upper lip anterior point and Esthetic line, LLE= Distance of lower lip anterior point and Esthetic line. B) Angular measurements; SNA= Sella.Nasion.A point, SNB= Sella.Nasion.B point, ANB= A point.Nasion.B point, SN.GoGn= Sella-Nasion.Gonion-Gnathion, SN.PP= Sella-Nasion.Palatal Plane, U1.SN= Long axis of upper incisor.Sella-Nasion, U1.PP= Long axis of upper incisor.Palatal Plane, U1.NA= Long axis of upper incisor.Nasion-A point, IMPA= long axis of lower incisor.Mandibular Plane, L1.NB= Long axis of lower incisor.Nasion-B point, I/I= Interincisal angle, NLA= Nasolabial angle.

The analysis required for the classification of skeletal malocclusion was carried out with Dolphin Imaging software^5^ (Fig 1A). Subsequently, the same cephalometric radiographs were loaded into the cloud-based WebCeph software^13^ (Fig 1B), and analyses were performed using AI. The cephalometric points used in the study are presented in Figure 2, and illustrations of the linear and angular measurements are presented in Figure 3. The subgrouping of the individuals included in the study according to the sagittal skeletal malocclusion classification was determined by skeletal Class I (0 < ANB < 4), Class II (ANB > 4), and Class III (ANB < 0) in the sagittal dimension.^5^ ^,^ ^14^ The cephalometric radiographs of 105 individuals (49 female, 17.30 ± 2.08 years; 56 male, 17.21 ± 1.68 years) aged 15 and over (35 skeletal Class I, 35 skeletal Class II, and 35 skeletal Class III) who applied to the Department of Orthodontics of Erciyes University with a request for orthodontic treatment were included in the study (Table 1). All radiographs to be used in the study were taken by the same technician, using the same cephalometry device (OP100; Instrumentarium, Tuusula; Finland) and the cephalostat of the device, with the Frankfurt plane parallel to the ground, the teeth in centric occlusion, and the lips in a resting position. The determination of cephalometric points for cephalometric analysis performed with the DM method was carried out by a single experienced orthodontist. In the cephalometric analysis performed with the web-based AI method, the cephalometric points were placed automatically by artificial intelligence, and the evaluation was provided without any intervention. Parameters of the different linear and angular hard and soft tissues measurements used in this study are listed below (Fig 3):

**Table 1:** Demographic distribution of individuals included in the study, organized by group.

	Class I			Class II			Class III			Total		
	n	Mean	SD	n	Mean	SD	n	Mean	SD	n	Mean	SD
Female	15	16.03	1.48	18	17.84	2.15	16	17.87	2.05	49	17.30	2.08
Male	20	16.72	1.28	17	17.23	2.28	19	17.71	1.32	56	17.21	1.68
Total	35	16.43	1.39	35	17.54	2.20	35	17.78	1.67	105	17.25	1.87

n= Number of subject. SD= Standard Deviation.

Linear measurements (mm)

» NA: the distance between Nasion and A point;
» NPog: the distance between Nasion and Pogonion;
» ANS-Me: the distance between Anterior Nasal Spine and Menton;
» CoA: the distance between Condylion and A point;
» CoGn: the distance between Condylion and Gnathion;
» U1-NA: the distance between the long axis of upper incisor and Nasion-A point;
» L1-APog: the distance between the long axis of lower incisor and A point-Pogonion;
» L1-NB: the distance between the long axis of lower incisor and Nasion-B point;
» ULE: the distance between the upper lip anterior point and Steiner’s Esthetic line;
» LLE: the distance between the lower lip anterior point and Steiner’s Esthetic line.

Angular measurements (degrees)

» SNA: Sella.Nasion.A point angle;
» SNB: Sella.Nasion.B point angle;
» ANB: A point.Nasion.B point angle;
» SN.GoGn: Sella-Nasion.Gonion-Gnathion angle;
» SN.PP: Sella-Nasion.Palatal Plane angle;
» U1.SN: Long axis of upper incisor.Sella-Nasion angle;
» U1.PP: Long axis of upper incisor.Palatale Plane angle;
» U1.NA: Long axis of upper incisor.Nasion-A point angle;
» IMPA: Long axis of lower incisor.mandibular plane angle;
» L1.NB: Long axis of lower incisor.Nasion-B point angle;
» I/I: Interincisal angle;
» NLA: Nasolabial angle.

### STATISTICAL ANALYSIS

The data obtained as a result of the study were recorded in Microsoft Excel (Microsoft Office, 365, Redmond, Washington, USA). A statistical evaluation of the data was performed on a computer using the Statistical Package for the Social Sciences (v. 24.0, IBM, Vermont, USA) analysis software. Shapiro-Wilk and Kolmogorov-Smirnov tests were used to determine whether the data showed a normal distribution. The paired *t*-test was used to evaluate the measurements obtained by DM and AI methods belonging to the same cephalometries. The One-Way ANOVA and *post-hoc* Tukey test were used for comparisons between malocclusion groups. The representation of the data is presented in the form of mean and standard deviation. The intraclass correlation coefficient (ICC) was used to evaluate the reliability between the two methods.^11^ The statistical significance value was defined as *p*< 0.05.

### METHOD ERROR

In order to evaluate the reliability of each of the cephalometric analysis methods, the measurements of 10 samples, randomly selected among all samples, were repeated two weeks after the first measurement by the same researcher for the DM method. A separate account was created for the AI method, and the radiographs were reloaded. The reliability analysis of the repeatability between the first and second measurements was evaluated using the ICC. Accordingly, the confidence interval for the DM method was found between 0.768 (95% confidence interval, lower bound) and 0.997 (95% confidence interval, upper bound), while it was between 0.940 (95% confidence interval, lower bound) and 0.999 (95% confidence interval, upper bound) for WebCeph.

## RESULTS

Demographic data indicating the age and gender information of the individuals included in this study are presented in Table 1.

The data of the measurements examined with the digitally drawn cephalometric radiograph method and the AI-based automatic measurement method are presented in Table 2. It was found a statistically significant difference (*p*<0.05) between the two methods for the following measurements: SNA, ANB, NA, Y-axis, SN.GoGn, SN.PP, ANS-Me, CoA, CoGn, U1.PP, U1-NA, IMPA, L1-NB, L1.NB, NLA and ULE. Among these, only Y-axis, SN.GoGn, U1-NA, and NLA parameters obtained higher values than WebCeph program in the measurements performed with Dolphin software, while WebCeph software presented higher values for other parameters (Table 2). 

**Table 2:** Evaluation of linear and angular measurements obtained from Dolphin Imaging software used for DM cephalometric analysis and WebCeph platform used for AI-based automated cephalometric analysis.

Variables	Dolphin				Webceph			Difference			p-value
	Mean	SD	Min	Max	Mean	SD	Min	Mean	SD	DP	
SNA (º)	80.67	4.34	67.00	90.70	81.98	3.29	74.04	89.34	-1.30	2.74	0.000
SNB (º)	79.08	5.25	70.10	95.20	78.93	4.05	70.72	90.78	0.15	2.45	0.527
ANB (º)	1.60	4.00	-7.20	8.70	3.05	3.35	-5.63	9.20	-1.45	1.46	0.000
NA (mm)	-0.88	3.96	-10.40	8.60	1.11	3.02	-8.42	6.84	-1.99	2.99	0.000
NPog (mm)	-2.68	8.14	-20.70	19.40	-1.72	7.21	-19.54	17.76	-0.95	5.30	0.068
Y-axis (º)	59.92	4.30	45.80	69.80	58.84	3.31	50.09	69.07	1.08	2.74	0.000
SN.GoGn (º)	32.51	6.75	15.90	54.30	31.31	5.35	18.28	41.26	1.19	3.55	0.001
SN.PP (º)	7.54	4.45	-8.20	17.30	9.04	2.57	3.93	15.79	-1.51	3.56	0.000
ANS-Me (mm)	65.74	5.49	52.60	78.20	71.25	5.71	54.57	82.11	-5.51	4.35	0.000
CoA (mm)	82.36	5.92	69.50	98.60	88.72	3.12	80.08	95.44	-6.36	5.74	0.000
CoGn (mm)	117.25	7.86	101.00	133.80	121.18	7.06	103.41	138.58	-3.93	7.69	0.000
U1.SN (º)	103.71	8.56	74.30	123.40	103.98	5.78	93.86	124.25	-0.28	6.07	0.642
U1.PP (º)	111.42	7.74	87.40	126.90	113.02	4.85	101.75	129.20	-1.60	5.77	0.005
U1-NA (mm)	4.52	3.15	-6.70	11.70	3.52	1.87	0.26	8.52	1.01	2.55	0.000
U1.NA (º)	23.11	8.55	-6.70	38.20	22.06	5.16	8.56	36.67	1.05	6.27	0.000
L1-APog (mm)	2.39	3.20	-7.20	10.30	2.18	2.62	-4.38	8.86	0.20	1.78	0.245
IMPA (º)	89.39	8.32	67.30	107.80	92.15	8.36	70.85	107.14	-2.76	4.11	0.000
L1-NB (mm)	4.29	2.65	-2.70	12.30	5.15	2.57	0.11	13.60	-0.87	1.43	0.000
L1.NB (º)	22.86	7.29	1.30	41.80	23.94	6.01	10.48	40.48	-1.09	5.46	0.044
I/I (º)	132.19	11.83	96.10	163.30	131.05	8.84	102.30	150.03	1.14	7.92	0.143
NLA (º)	107.46	12.92	53.90	140.70	97.05	8.49	72.17	116.16	10.40	9.74	0.000
ULE (mm)	-4.64	2.95	-12.80	3.70	-3.64	3.06	-9.65	4.40	-1.00	2.13	0.000
LLE (mm)	-2.27	3.24	-10.80	4.90	-2.41	2.86	-9.33	5.54	0.13	2.02	0.500

NPog= Nasion?Pogonion; NA= Nasion?A point; ANS-Me= Anterior Nasal Spine?Menton; CoA= Condylion?A point; CoGn= Condylion?Gnathion; U1-NA= Long axis of upper incisor?Nasion-A point; L1-APog= Long axis of lower incisor?A point-Pogonion; L1-NB= Long axis of lower incisor?Nasion-B point; ULE= Distance of upper lip anterior point and Esthetic line; LLE= Distance of lower lip anterior point and Esthetic line; SNA= Sella.Nasion.A point; SNB= Sella.Nasion.B point; ANB= A point.Nasion.B point; SN.GoGn= Sella-Nasion.Gonion-Gnathion; SN.PP= Sella-Nasion.Palatal Plane; U1.SN= Long axis of upper incisor.Sella-Nasion; U1.PP= Long axis of upper incisor.Palatal Plane; U1.NA= Long axis of upper incisor.Nasion-A point; IMPA= long axis of lower incisor.Mandibular Plane; L1.NB= Long axis of lower incisor.Nasion-B point; I/I= Interincisal angle; NLA= Nasolabial Angle; (º)= Degree (for angle); (mm)= millimeter (for distance); SD= Standard Deviation; Min= Minimum; Max= Maximum. Statistical significance degree= p < 0.05.

Skeletal malocclusion classification was initially performed using the analysis performed with Dolphin software (Table 3). In each malocclusion Class, Dolphin digital software and WebCeph AI software were compared. It was determined that the parameters differing in all three malocclusion groups for both analysis methods were ANB, NA, ANS-Me, CoA, CoGn, L1-APog, IMPA, NLA (Table 3, *p*<* *0.05). Only the LLE parameter differed in Class I. It was determined that parameters differing only in the Class II group were SNB, SN.GoGn, and I/I (Table 3, *p*<* *0.05). It was determined that the parameter differing only in the Class III group was U1/NA (Table 3, *p*<0.05). 

**Table 3:** Evaluation and comparison of linear and angular measurements obtained from Dolphin Imaging software used for DM cephalometric analysis and WebCeph platform used for AI-based automated cephalometric analysis, according to skeletal malocclusion classes.

	Class I					Class II					Class III				
	Dolphin		WebCeph		p value	Dolphin		WebCeph		p value	Dolphin		WebCeph		p value
	Mean	SD	Mean	SD		Mean	SD	Mean	SD		Mean	SD	Mean	SD	
SNA (º)	80.95	4.90	81.73	3.52	0.102	82.08	2.11	83.30	2.09	0.000	78.98	4.90	80.89	3.63	0.002
SNB (º)	78.97	5.34	78.23	4.06	0.075	76.13	2.22	77.01	2.15	0.005	82.13	5.69	81.54	4.20	0.221
ANB (º)	1.98	1.00	3.49	1.45	0.000	5.95	1.23	6.30	1.31	0.048	-3.13	1.95	-0.64	2.32	0.000
NA (mm)	-1.43	3.62	0.62	3.06	0.000	1.66	3.07	2.79	2.39	0.014	-2.87	3.81	-0.08	2.86	0.000
NPog (mm)	-4.27	7.41	-3.61	6.22	0.436	-5.94	6.07	-5.51	4.79	0.607	2.17	8.54	3.95	6.75	0.092
Y-axis (º)	61.06	4.30	59.71	3.28	0.004	60.87	3.20	59.80	2.75	0.018	57.82	4.58	57.00	3.16	0.135
SN.GoGn (º)	31.62	6.36	31.29	5.74	0.553	33.08	6.36	31.28	5.37	0.000	32.82	7.57	31.37	5.07	0.062
SN.PP (º)	6.57	4.42	8.90	2.99	0.000	8.95	4.19	8.96	2.31	0.992	7.08	4.51	9.27	2.43	0.001
ANS-Me (mm)	66.35	6.67	71.80	5.98	0.000	66.01	5.06	70.73	5.64	0.000	64.88	4.58	71.24	5.63	0.000
CoA (mm)	81.87	5.10	88.06	2.82	0.000	85.49	5.00	90.14	3.06	0.000	79.73	6.24	87.96	3.07	0.000
CoGN (mm)	116.67	7.89	120.01	5.36	0.011	114.59	5.93	117.03	5.60	0.017	120.48	8.55	126.50	6.63	0.001
U1.SN (º)	103.13	7.12	102.99	5.90	0.845	100.50	10.36	103.14	5.39	0.046	107.49	6.36	105.83	5.77	0.000
U1.PP (º)	110.10	7.03	111.86	4.94	0.028	109.36	9.08	112.10	4.47	0.019	114.79	5.80	115.10	4.58	0.760
U1-NA (mm)	4.65	2.34	3.23	1.79	0.000	2.65	3.82	3.27	2.08	0.170	6.27	1.89	4.05	1.64	0.000
U1.NA (º)	22.35	6.04	21.33	4.57	0.235	18.45	10.25	19.85	5.31	0.244	28.53	5.37	25.01	4.22	0.001
L1-APog	2.04	2.00	1.68	2.20	0.026	1.09	3.50	2.12	2.85	0.002	4.03	3.22	2.75	2.72	0.000
IMPA (º)	90.15	4.98	92.58	4.76	0.000	95.08	7.39	99.45	5.31	0.000	82.93	7.47	84.41	6.80	0.044
L1-NB (mm)	4.10	1.80	4.99	1.94	0.000	5.72	3.15	7.05	2.58	0.000	3.04	2.15	3.43	1.73	0.122
L1.NB (º)	22.62	5.41	23.89	4.06	0.128	26.46	8.96	29.26	4.76	0.018	19.49	5.27	18.69	3.73	0.265
I/I (º)	132.73	8.19	131.42	7.29	0.130	128.35	15.68	124.65	8.48	0.031	135.50	9.41	137.08	5.86	0.225
NLA (º)	110.73	11.45	99.95	7.41	0.000	110.00	11.83	99.56	5.28	0.000	101.64	13.67	91.65	9.62	0.000
ULE (mm)	-4.98	2.03	-3.54	1.91	0.000	-2.66	2.15	-0.97	2.17	0.000	-6.28	3.30	-6.41	2.25	0.759
LLE (mm)	-2.49	2.29	-2.94	2.53	0.046	-0.75	2.72	-0.73	2.58	0.932	-3.58	3.90	-3.55	2.73	0.943

NPog= Nasion?Pogonion; NA= Nasion?A point; ANS-Me= Anterior Nasal Spine?Menton; CoA= Condylion?A point; CoGn= Condylion?Gnathion; U1-NA= Long axis of upper incisor?Nasion-A point; L1-APog= Long axis of lower incisor?A point-Pogonion; L1-NB= Long axis of lower incisor?Nasion-B point; ULE= Distance of upper lip anterior point and Esthetic line; LLE= Distance of lower lip anterior point and Esthetic line; SNA= Sella.Nasion.A point; SNB= Sella.Nasion.B point; ANB= A point.Nasion.B point; SN.GoGn= Sella-Nasion.Gonion-Gnathion; SN.PP= Sella-Nasion.Palatal Plane; U1.SN= Long axis of upper incisor.Sella-Nasion; U1.PP= Long axis of upper incisor.Palatal Plane; U1.NA= Long axis of upper incisor.Nasion-A point; IMPA= long axis of lower incisor.Mandibular Plane; L1.NB= Long axis of lower incisor.Nasion-B point; I/I= Interincisal angle; NLA= Nasolabial angle; (º)= Degree (for angle); (mm)= millimeter (for distance); SD= Standard Deviation; Min= Minimum; Max= Maximum. Statistical significance degree= *p* < 0.05.

The relationship between each skeletal malocclusions and the difference between Dolphin software and WebCeph software for each parameter is presented in Table 4. It was determined that there was a significant difference between all three skeletal Class groups for the parameters SNB, ANB, SN.PP, U1.SN, U1-NA, U1.NA, L1-APog, IMPA, L1-NB, and ULE (Table 4, *p*<* *0.05). In a paired comparison of the groups for the ANB parameter, it was found that there were differences in all three groups (Table 4). 

**Table 4:** Evaluation and comparison of the differences between the measurements obtained after the cephalometric analysis performed by the DM method and the AI-based automated method, according to the skeletal malocclusion groups.

	Class I		Class II		Class III		p value	Binary Comparisons		
	Mean	SD	Mean	SD	Mean	SD		I-II	I-III	II-III
SNA (º)	-0.78	2.74	-1.22	1.86	-1.91	3.37	0.321	0.771	0.195	0.545
SNB (º)	0.74	2.38	-0.88	1.73	0.60	2.82	0.008	0.013	0.964	0.027
ANB (º)	-1.51	1.17	-0.35	1.04	-2.49	1.30	0.000	0.000	0.002	0.000
NA (mm)	-2.04	2.99	-1.13	2.57	-2.79	3.23	0.054	0.396	0.538	0.051
NPog	-0.66	4.93	-0.43	4.90	-1.78	6.06	0.731	0.983	0.653	0.541
Y-axis (º)	1.35	2.56	1.06	2.53	0.82	3.15	0.590	0.904	0.701	0.924
SN.GoGn (º)	0.33	3.30	1.80	2.59	1.45	4.45	0.257	0.198	0.385	0.912
SN.PP (º)	-2.33	2.86	-0.01	3.64	-2.19	3.74	0.004	0.002	0.760	0.006
ANS-Me (mm)	-5.45	4.91	-4.72	3.53	-6.36	4.45	0.292	0.762	0.660	0.261
CoA (mm)	-6.20	5.73	-4.65	4.72	-8.23	6.26	0.122	0.480	0.289	0.064
CoGN (mm)	-3.34	7.35	-2.44	5.77	-6.02	9.29	0.128	0.873	0.308	0.125
U1.SN (º)	0.14	4.31	-2.63	7.53	1.66	5.27	0.010	0.122	0.527	0.008
U1.PP (º)	-1.76	4.54	-2.74	6.58	-0.31	5.90	0.301	0.756	0.541	0.184
U1-NA (mm)	1.43	2.17	-0.62	2.62	2.21	1.97	0.000	0.001	0.318	0.000
U1.NA (º)	1.02	4.98	-1.40	7.01	3.53	5.82	0.004	0.470	0.002	0.015
L1-APog	0.36	0.91	-1.03	1.79	1.28	1.70	0.000	0.003	0.062	0.000
IMPA (º)	-2.43	3.45	-4.37	4.23	-1.48	4.18	0.042	0.184	1.000	0.048
L1-NB (mm)	-0.89	0.98	-1.33	1.65	-0.39	1.45	0.030	1.000	0.228	0.028
L1.NB (º)	-1.27	4.80	-2.79	6.64	0.80	4.15	0.057	0.454	0.239	0.055
I/I (º)	1.31	4.99	3.70	9.74	-1.58	7.59	0.121	0.398	0.262	0.054
NLA (º)	10.78	10.61	10.44	9.11	9.99	9.71	0.931	0.989	0.938	0.979
ULE (mm)	-1.44	1.74	-1.69	1.62	0.13	2.48	0.000	0.858	0.004	0.001
LLE (mm)	0.46	1.30	-0.02	1.47	-0.04	2.90	0.492	0.587	0.568	1.000

NPog= Nasion?Pogonion; NA= Nasion?A point; ANS-Me= Anterior Nasal Spine?Menton; CoA= Condylion?A point; CoGn= Condylion?Gnathion; U1-NA= Long axis of upper incisor?Nasion-A point; L1-APog= Long axis of lower incisor?A point-Pogonion; L1-NB= Long axis of lower incisor?Nasion-B point; ULE= Distance of upper lip anterior point and Esthetic line; LLE= Distance of lower lip anterior point and Esthetic line; SNA= Sella.Nasion.A point; SNB= Sella.Nasion.B point; ANB= A point.Nasion.B point; SN.GoGn= Sella-Nasion.Gonion-Gnathion; SN.PP= Sella-Nasion.Palatal Plane; U1.SN= Long axis of upper incisor.Sella-Nasion; U1.PP= Long axis of upper incisor.Palatal Plane; U1.NA= Long axis of upper incisor.Nasion-A point; IMPA= long axis of lower incisor.Mandibular Plane; L1.NB= Long axis of lower incisor.Nasion-B point; I/I= Interincisal angle; NLA= Nasolabial angle; (º)= Degree (for angle); (mm)= millimeter (for distance); SD= Standard Deviation; Min= Minimum; Max= Maximum. Statistical significance degree= *p* < 0.05.

An evaluation of the reliability between the two cephalometric analyzes is presented in Table 5. Accordingly, it was determined that the CoA and CoGn parameters showed low values (ICC* *<* *0.50, Table 5).

**Table 5:** Evaluation and comparison of the reliability of DM method and the AI-based automated method.

	Class I			Class II			Class III			Total		
	ICC*	95% CI		ICC*	95% CI		ICC*	95% CI		ICC*	95% CI	
		Upper Bound	Lower Bound		Upper Bound	Lower Bound		Upper Bound	Lower Bound		Upper Bound	Lower Bound
SNA (º)	0.885	0.774	0.942	0.757	0.518	0.877	0.820	0.643	0.909	0.855	0.786	0.901
SNB (º)	0.933	0.867	0.966	0.814	0.632	0.906	0.913	0.828	0.956	0.927	0.892	0.950
ANB (º)	0.715	0.435	0.826	0.801	0.605	0.899	0.899	0.799	0.949	0.959	0.940	0.972
NA (mm)	0.752	0.509	0.875	0.722	0.449	0.860	0.702	0.410	0.850	0.780	0.677	0.851
NPog (mm)	0.851	0.705	0.925	0.749	0.503	0.873	0.871	0.637	0.908	0.865	0.801	0.908
Y-axis (º)	0.874	0.751	0.936	0.782	0.567	0.890	0.809	0.622	0.904	0.854	0.785	0.901
SN.GoGn (º)	0.920	0.841	0.960	0.949	0.900	0.974	0.865	0.732	0.932	0.907	0.863	0.937
SN.PP (º)	0.832	0.668	0.915	0.595	0.197	0.796	0.637	0.281	0.817	0.684	0.535	0.785
ANS-Me (mm)	0.823	0.649	0.911	0.878	0.759	0.939	0.769	0.542	0.883	0.823	0.739	0.880
CoA (mm)	0.061	-0.861	0.526	0.521	0.051	0.758	0.319	-0.349	0.656	0.418	0.144	0.605
CoGN (mm)	0.578	0.164	0.787	0.666	0.338	0.831	0.416	-0.157	0.705	0.640	0.470	0.755
U1.SN (º)	0.878	0.759	0.939	0.737	0.480	0.867	0.769	0.541	0.883	0.791	0.693	0.858
U1.PP (º)	0.838	0.679	0.918	0.732	0.469	0.865	0.532	0.073	0.764	0.751	0.634	0.831
U1-NA (mm)	0.628	0.264	0.812	0.780	0.563	0.889	0.556	0.120	0.776	0.682	0.532	0.784
U1.NA (º)	0.724	0.453	0.861	0.774	0.552	0.886	0.429	-0.131	0.712	0.754	0.638	0.833
L1-APog (mm)	0.951	0.903	0.975	0.915	0.831	0.957	0.911	0.824	0.955	0.898	0.850	0.931
IMPA (º)	0.856	0.716	0.928	0.879	0.760	0.939	0.906	0.814	0.953	0.935	0.905	0.956
L1-NB (mm)	0.927	0.855	0.963	0.911	0.823	0.955	0.838	0.680	0.918	0.919	0.881	0.945
L1.NB (º)	0.663	0.333	0.830	0.727	0.460	0.862	0.740	0.484	0.869	0.800	0.705	0.864
I/I (º)	0.885	0.771	0.942	0.825	0.652	0.911	0.694	0.393	0.845	0.832	0.753	0.886
NLA (º)	0.566	0.140	0.781	0.671	0.349	0.834	0.797	0.598	0.898	0.752	0.635	0.832
ULE (mm)	0.759	0.522	0.878	0.835	0.674	0.917	0.761	0.526	0.879	0.857	0.789	0.903
LLE (mm)	0.922	0.845	0.960	0.918	0.837	0.958	0.772	0.548	0.885	0.878	0.820	0.917

NPog= Nasion?Pogonion; NA= Nasion?A point; ANS-Me= Anterior Nasal Spine?Menton; CoA= Condylion?A point; CoGn= Condylion?Gnathion; U1-NA= Long axis of upper incisor?Nasion-A point; L1-APog= Long axis of lower incisor?A point-Pogonion; L1-NB= Long axis of lower incisor?Nasion-B point; ULE= Distance of upper lip anterior point and Esthetic line; LLE= Distance of lower lip anterior point and Esthetic line; SNA= Sella.Nasion.A point; SNB= Sella.Nasion.B point; ANB= A point.Nasion.B point; SN.GoGn= Sella-Nasion.Gonion-Gnathion; SN.PP= Sella-Nasion.Palatal Plane; U1.SN= Long axis of upper incisor.Sella-Nasion; U1.PP= Long axis of upper incisor.Palatal Plane; U1.NA= Long axis of upper incisor.Nasion-A point; IMPA= long axis of lower incisor.Mandibular Plane; L1.NB= Long axis of lower incisor.Nasion-B point; I/I= Interincisal angle; NLA= Nasolabial angle; (º)= degree (for angle); (mm)= millimeter (for distance); CI= Confidence Interval. ICC= Intraclass Correlation coefficient (Cronbach’ Alpha).

## DISCUSSION

This study, which has the quality of guiding future studies, aimed to investigate the relationship between and reliability of DM and AI-based cephalometric analysis methods, according to skeletal malocclusion classes in the sagittal dimension. In his study, Alqahtani^6^ noted that the cloud-based websites that support the cephalometric analysis method will be a practical tool because they are fast, make storage easy, require no installation, and are easily accessible on all website platforms.

In the study conducted by Kim et al,^15^ it was reported that an automatic cephalometric analysis created with deep learning can produce very realistic results. However, in this study, only randomly selected radiographs were evaluated, and no specific evaluation was made for skeletal malocclusions caused by different positions of the mandible and maxilla. In a study conducted by Nishimoto et al,^7^ it was stated that artificial intelligence with deep learning could be used to mark anatomical points in cephalometric radiographs. As recommended by Nishimoto et al,^7^ the cephalometric measurement differences between various malocclusion groups were evaluated, and it was determined that AI-based systems still need improvements. In a study conducted by Alqahtani,^6^ it was determined that the AI-based online cephalometric analysis method has high reproducibility and practical use. Therefore, the usability of this method in the context of different types of malocclusion was examined in the present study.^6^ However, these systems are still in the development phase, and their use in more specific areas should be investigated. Furthermore, it has been stated in the literature that the cephalometric drawings performed manually can differ between professionals as well as between repeated drawings of the same professional.^16^ ^,^ ^17^ For these reasons, the importance of AI-based automatic systems in reducing application errors is increasing. In order to contribute to these studies, it was found that the measurements used in cephalometric analysis may differ when grouping was made according to skeletal malocclusion classes in the present study. In the study of Yu et al,^18^ it was stated that the precision and accuracy of AI-based automatic skeletal malocclusion classification were high. However, the analyses for determining the skeletal Class were not mentioned. In the present study, it was determined that AI-based automatic cephalometric analysis can be used for different measurements, but still requires improvement. Based on the present results, it can be concluded that when evaluating cephalometric analysis for deep learning, more samples of different skeletal malocclusions should be evaluated.

In the present study, except for SNB, NPog, U1.SN, U1.NA, L1-APog, I/I, and LLE parameters, all other parameters presented significant differences between the DM and AI methods. However, these parameters may also differ between skeletal malocclusion groups. The correct determination of skeletal cephalometric parameters, which is the basic standard in the treatment of skeletal malocclusions, is essential for ideal treatment.^19^ When the reliability levels of the two methods were compared and evaluated, it was determined that both methods are suitable for orthodontic analysis.^5^ ^,^ ^8^ ^,^ ^13^ In this study, only the CoA and CoGn parameters had low reliability between methods. This was thought to be due to the difficulty of determining the condylion (Co) point.^20^ These findings indicate that, unlike the long-standing DM method,^2^ ^,^ ^5^ ^,^ ^12^ ^,^ ^19^ the AI-based automatic method still requires further development.

## CONCLUSION

Artificial intelligence-based cephalometric analysis methods can both accelerate and facilitate orthodontic treatment planning, thanks to easy archiving and automatic processes. The present study has shown that there are significant differences between the AI-based automatic method and the DM method in most cephalometric analysis measurements. Therefore, AI-based analysis needs further development and more testing in different malocclusion groups, which may change orthodontic treatment planning. The relative measurement reliability between the two techniques was found to be high, except for the CoA and CoGn measurements. There are significant differences between the measurement quantities in the two techniques, and the AI-based technique needs to be developed in these aspects. Although there are statistically significant differences in the measurements obtained between the two methods, the authors believe that there is no “clinically significant” difference between the methods to ensure a rapid preliminary assessment of orthodontic treatment planning. It is undeniable that rapidly developing AI-based technology procedures will bring significant changes both in dental and medical contexts in the future.
